# Controllable Pseudospin Topological Add-Drop Filter Based on Magnetic–Optical Photonic Crystals

**DOI:** 10.3390/nano14110919

**Published:** 2024-05-23

**Authors:** Chao Yan, Yuhao Huang, Zhi-Yuan Li, Wenyao Liang

**Affiliations:** School of Physics and Optoelectronics, South China University of Technology, Guangzhou 510641, China

**Keywords:** magnetic–optical photonic crystals, photonic crystal ring resonator, add-drop filter, pseudospin states

## Abstract

We propose a controllable topological add-drop filter based on magnetic–optical photonic crystals. This add-drop filter is composed of two straight waveguides and a hexagonal photonic crystal ring resonator. The waveguide and ring resonator are constructed by three different honeycomb magnetic–optical photonic crystals. The expanded lattice is applied with an external magnetic field so that it breaks time-reversal symmetry and the analogous quantum spin Hall effect simultaneously. While the standard one and the compressed one are not magnetized and trivial, the straight waveguide supports pseudospin-down (or pseudospin-up) one-way states when the expanded lattice is applied with an external magnetic field of +*H* (or −*H*). The ring resonator possesses multiple resonant modes which can be divided into travelling modes and standing modes. By using the travelling modes, we have demonstrated the function of the add-drop filter and realized the output port control by changing the direction of the magnetic field. Moreover, a large tunable power ratio from near 0 to 52.6 is achieved by adjusting the strength of the external magnetic field. The structure has strong robustness against defects due to the topological protection property. These results have potential in wavelength division multiplexing systems and integrated topological optical devices.

## 1. Introduction

Photonic crystals (PCs) provide excellent conditions to manipulate light and electromagnetic (EM) waves on the subwavelength scale [1,2]. In ordinary dielectric PCs, photons are easy to be backscattered by defects and impurities. In 2008, by introducing an external magnetic field (EMF) to the magneto-optical photonic crystal (MOPC) to break time-reversal symmetry (TRS), Haldane and Raghu found that the Dirac point of the band structure is broken down to open a topological bandgap which supports the unidirectional topological edge state. For these unidirectional edge states, EM waves propagate unidirectionally with strong robustness against backscattering and defects. Subsequently, inspired by these works, researchers proposed various photonic analogies to the quantum Hall (QH) effect, quantum spin Hall (QSH) effect, and quantum valley Hall (QVH) effect [3,4,5,6,7].

In 2015, WU et al. [8] proposed that the pseudospin-dependent helical edge state can be reached by compressing and expanding the honeycomb lattice of PCs. Two honeycomb lattices have different Chern numbers, which cause the pseudospin-up (or pseudospin-down) state when they are put together. In addition, various topological devices such as the pseudospin beam splitter [9], pseudospin polarization topological line defect [10], and unidirectional waveguide based on dielectric materials [11] have been realized. Generally, pseudospin waveguides are constructed by a topological PC and a trivial PC. They only support edge states to propagate along the interface between the two PCs, meaning that its waveguide available area is very narrow, and space utilization is limited. Recently, three kinds of methods have been proposed to achieve large-area one-way transmissions in a three-layer heterogeneous structure by the photonic analogue to QH effect [12], QVH effect [13,14], and QSH effect [15,16], which provide a feasible idea to broaden the waveguide area for effective transmission. Furthermore, according to the research of Yu et al. [16], pseudospin-field-dependent waveguide transmission can be realized and regulated by applying magnetic fields in a heterogeneous structure.

In recent years, photonic crystal ring resonators (PCRRs) have attracted great attention due to their excellent characteristics such as low loss, high quality factor, as well as flexible configurations. Because of their efficient resonant coupling nature, various PCRR-based optical devices, such as ultra-high-Q optical filter [17] and topological ADF [18,19,20], have been proposed. Especially noteworthy is an add-drop filter (ADF), which allows the transfer of one or several frequency channels from the bus waveguide through a resonator system to the drop waveguide without disturbing the other channels, playing an important role in many application fields, such as wavelength division multiplexing systems. Earlier ADFs were based on microcavity, and they usually support only one resonant mode. Later, ADFs based on dielectric PCRR with different shapes were designed [21,22,23,24,25], which would generate backscattering due to surface roughness and affect performance. Very recently, Tang et al. proposed topological multichannel MOPC-based ADFs, which possess strong robustness which results from the broken TRS. Inspired by these works, it is highly desired to realize a topological ADF with output port control and an adjustable power ratio by using pseudospin-dependent MOPC waveguide and PCRR together.

In this work, we propose a controllable topological ADF which is constructed by two straight MOPC waveguides and a hexagonal PCRR. The MOPC waveguide supports the pseudospin-dependent state, while the PCRR possesses multiple resonant modes which are divided into travelling modes and standing modes. Based on the coupling effect between the pseudospin waveguide state and the travelling mode, we have realized the function of ADF. Additionally, the output port and power ratio control of the ADF have been achieved by modifying the direction and strength of the EMF, respectively. This topological device has strong robustness against obstacles. Our work may provide feasible ideas for designing efficient topological optical devices and further understand wavelength division multiplexing systems.

## 2. Materials and Methods

### 2.1. Basic Honeycomb PC and Band Structure Analyses

**Figure 1:**
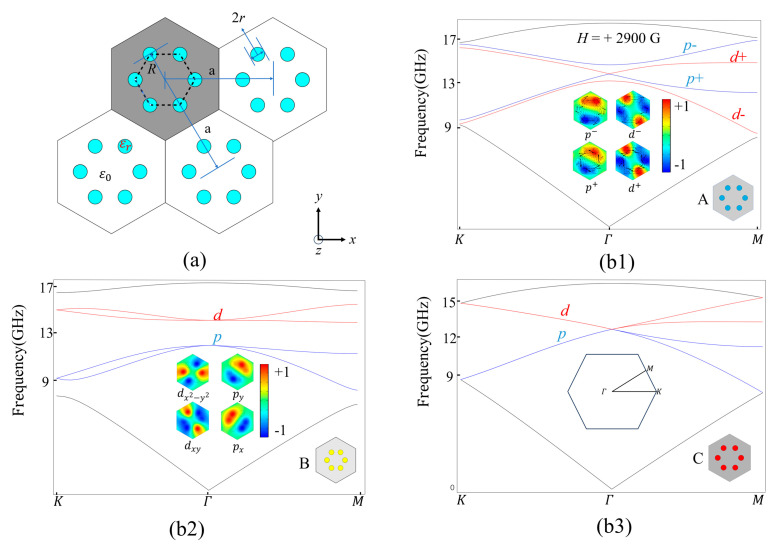
The basic structure of the honeycomb lattice and the band structure: (**a**) the parameter definitions of the honeycomb lattice; (**b1**–**b3**) the band structure of expanded lattice A (blue), compressed lattice B (yellow), and standard lattice C (red). The eigenfields in lattices A and B are showed in (**b1**,**b2**).

Figure 1a shows that the basic MOPC model considered here is a triangular lattice of hexagonal clusters. Each hexagonal cluster consists of six yttrium iron garnet (YIG) rods, and it has C6v symmetry. The grey region represents the primitive cell, and the dashed black hexagon labels the artificial atom. The lattice constant of the basic MOPC is *a* = 1.15 cm. The dielectric constant and radius of each YIG rod are $\varepsilon_{r}$ = 15 and *r* = 0.09*a*. The rods are arranged in air, whose dielectric constant is 1. The distance between the centers of the YIG rod and the center of its lattice is *R*, as denoted in Figure 1a. We design three different lattices, i.e., expanded lattice A (Figure 1(b1). We mark it with a blue color further in the paper), compressed lattice B (Figure 1(b2). We mark it with a yellow color further in the paper), and standard honeycomb lattice C (Figure 1(b3). We mark it with the blue color further in the paper), with *a*/*R* = 2.88, 3.68, and 3, respectively, as shown by the insets of Figure 1(b1–b3). Lattices B and C are non-magnetized, while lattice A is applied with an EMF of +H=2900 G along the +*z* (or −*z*) axis, which causes the permeability of the YIG to become the following tensor [5,26]

(1) $$\hat{\mu }=\left(\begin{array}{ccc} \mu_{r} & -j\mu_{k} & 0\\ j\mu_{k} & \mu_{r} & 0\\ 0 & 0 & 1 \end{array}\right).$$

where *μ_r_* = 1 + *ω_m_*(*ω*_0_ + *jαω*)/[(*ω*_0_ + *jαω*)^2^ − *ω*^2^], *μ_k_* = *ωω_m_*/[(*ω*_0_ + *jαω*)^2^ − *ω*^2^]. In the above formula, *ω_m_* = 2*πγM*_0_ is the characteristic frequency, *ω*_0_ = 2*πγH*_0_ is the resonant frequency, *γ* = 2.8 MHz/Oe is the magnetic rotatory ratio, the saturation magnetization intensity is *M*_0_ = 1780 G, and *α =* 0.0003*j* is the damping coefficient that can be ignored. We use the commercial software COMSOL MULTIPHYSICS 5.6 to calculate the band structures and all the simulations in the frequency domain. Details specific to the computational methods are shown in Table A1 in Appendix A. It is noted that TM mode (*H_x_*, *H_y_*, and *E_z_* ≠ 0) can couple with the anisotropic permeability of the YIG due to the EMF along the *z* axis, while TE mode (*E_x_*, *E_y_*, and *H_z_* ≠ 0) does not. Therefore, only TM mode is considered in this work. In addition, to avoid the appearance of TE mode and confine the EM wave in *xoy* plane, the structure should have a thickness less than 1*a* and be covered between two metal plates on the bottom and the top. Under these conditions, only TM mode is supported, and we can use a 2D model to carry out the simulations for simplicity.

The band structures for lattices A, B, and C are calculated by the finite element method and shown in Figure 1(b1–b3). For the standard lattice C (*a*/*R* = 3) without an external magnetic field, its band structure has a double Dirac cone at Γ, as shown in Figure 1(b3). When the lattice is compressed (lattice B with *a*/*R* = 3.68), the two Dirac cones are separated into two pairs of two-fold degenerate p (blue) and d (red) states and create a band gap of [11.99, 14.08] (GHz). However, lattice B does not have band inversion, meaning that it is a trivial MOPC. The eigenmode functions at the Dirac cone can be classified into $p_{x}$, $p_{y}$, $d_{xy}$, $d_{x^{2}-y^{2}}$ based on spatial parities and pseudospin basis states, and the field patterns of degenerate *p* and *d* states are shown on the right of Figure 1(b2). Differently, for the expanded lattice A (*a*/*R* = 2.88) with +H=2900 G, Figure 1(b1) displays that it has band inversion, indicating that it is a nontrivial MOPC. Due to the broken TRS and space inversion symmetry simultaneously, band inversion occurs to create four kinds of eigenfields [5,11,27], i.e., $p_{\pm }=p_{x}\pm ip_{y}$ and $d_{\pm }=d_{xy}\pm id_{x^{2}-y^{2}}$. The field patterns of $p_{\pm }$ and $d_{\pm }$ states and the corresponding Poynting vectors $\vec{S}=R_{e}\left[\vec{E}\times \vec{H*}\right]/2$ are shown on the right of Figure 1(b1). The clockwise and counterclockwise patterns of the Poynting vectors in lattice A represent the pseudospin-down and pseudospin-up states, respectively [9,28].

We can use the spin Chern number to characterize the properties of the band structure [29,30], and this can be written as follows:

(2) $$C_{\pm }=\pm \left[sgn\left(B\right)+sgn(M_{0}\pm g)\right]/2$$

where *B* and *M*_0_ are model parameters defined by the coupling coefficients and *g* is the strength of a uniform exchange field [31]. For the cases of +H and −H, the spin Chern number of lattice A (*a*/*R* = 2.88) is $C_{\pm }=\left(0,-1\right)\;and\;C_{\pm }=\left(1,\;0\right)$, respectively, where the signs of + and − in $C_{\pm }$ correspond to pseudospin-up and -down components [16], incicating that lattice A is a topological MOPC. However, the spin Chern number of lattice B (*a*/*R* = 3.68) with no EMF is $C_{\pm }=0$, meaning that it is a trivial MOPC. Additionally, the presence of the Dirac cone of lattice C (*a*/*R* = 3) is not broken, meaning that it is also a trivial MOPC. These results are consistent with the band inversion analyses conducted previously.

### 2.2. Projected Band Structure of ACB Sandwiched Waveguide

**Figure 2:**
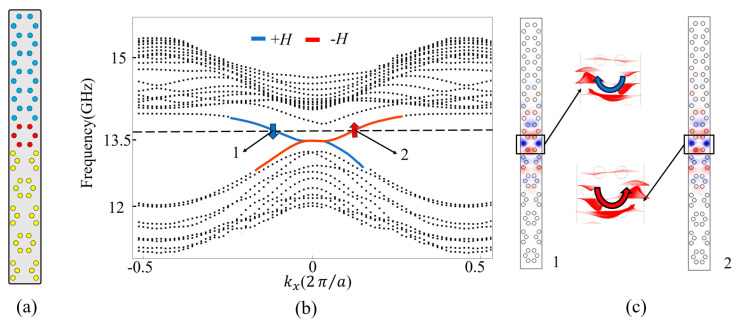
(**a**) Schematic diagram of the supercell structure formed by lattices A, B, and C; (**b**) projected band of the supercell. The red and blue arrows represent pseudospin-up and pseudospin-down states, respectively; (**c**) the eigenfield distributions and time-averaged Poynting vectors at points 1 and 2.

Now, we further construct a heterostructure waveguide by sandwiching lattice C between A and B (i.e., the ACB waveguide), and its supercell is shown in Figure 2b. The layer numbers of lattices A, C, and B are 6, 1, and 6, respectively. Figure 2a shows the projected band structures along the *k_x_* direction when lattice A is applied with +*H* = 2900 G or −*H* = −2900 G, respectively. It is found that there exists a bandgap from 13.25 to 13.62 GHz, and the red (or blue) solid line within the bandgap represents the waveguide state when −*H* (or +*H*) is applied to lattice A. Such red and blue curves have negative and positive slopes, respectively, meaning that they have opposite velocities. To analyze their propagation characteristics, a typical frequency *ω_s_* = 13.599 GHz intersecting with the red and blue curves at points 1 (+*H*) and 2 (−*H*) is adopted. From the eigenfields and time-averaged Poynting vectors at points 1 and 2 [Figure 2c], one can find that the blue and red curves correspond to pseudospin-down (clockwise pattern) and pseudospin-up (counterclockwise pattern) states, respectively. Therefore, due to the combined action of broken TRS and spatial inversion symmetry [16], the designed waveguide with +H/−H supports pseudospin-down leftwards/pseudospin-up rightwards one-way states, respectively.

### 2.3. Hexagonal Topological PCRR

**Figure 3:**
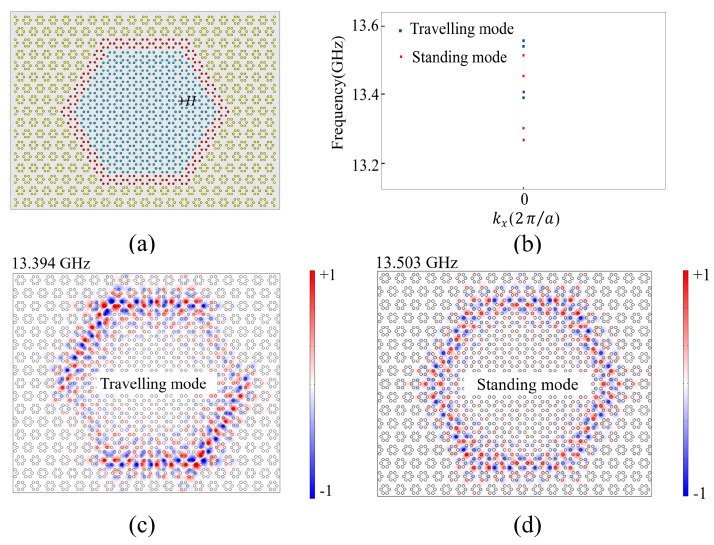
(**a**) The structure of the ring resonator; inside is the expanded lattice with an EMF +*H*; (**b**) Resonant frequencies of the ring resonator in topological band gap; (**c**,**d**) The$\;E_{z}$ field distributions of the travelling mode at frequency of 13.394 GHz and the standing mode at frequency of 13.503 GHz in the topological bandgap, respectively.

Next, we study the properties of the hexagonal PCRR depicted in Figure 3a. The PCRR is composed of three parts: inside part (lattice A), middle part (lattice C), and outside part (lattice B), all of which have the same parameters as discussed previously. Only inside part A is applied with +*H* along the *+z* axis.

We have calculated the resonant modes of the PCRR by the finite element method. The results in Figure 3b show that the PCRR supports multiple resonant modes within the topological band gap. Due to the six 120° corners in the PCRR, the degeneracy of a pair of cavity modes is lifted to form a pair of new modes called the traveling mode and standing mode (Figure 3b). Figure 3c,d present the$\;E_{z}$ field distributions of two typical modes oscillating at 13.394 GHz (travelling mode) and 13.503 GHz (standing mode). In order to understand the difference between the travelling mode and standing mode clearly, we provide two dynamic GIF figures as Figure S1 in Supplementary Materials. The dynamic GIF figures clearly demonstrate the evolution of the $E_{z}$ field distribution when its phase varies from 0 to 2π. One can find that the *E_z_* field flows clockwise along the boundary of the PCRR for the travelling mode at 13.394 GHz. Differently, for the standing mode at 13.503 GHz, the EM wave cannot flow and is localized at the boundary of the PCRR. It should be noted that in the topological bandgap, there are several groups of travelling modes and standing modes with similar properties [32,33] which play a major role in the coupling phenomenon between the topological waveguide and the PCRR [34,35].

### 2.4. Single Waveguide Coupling with PCRR

**Figure 4:**
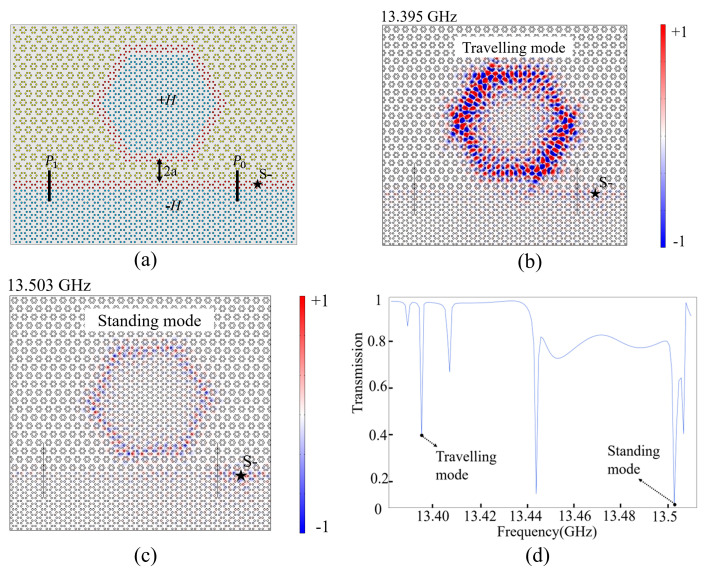
(**a**) Schematic diagram of single waveguide channel coupled with a PCRR. (**b**,**c**) $E_{z}$ field distributions at 13.395 GHz (travelling mode) and 13.503 GHz (standing mode). (**d**) Normalized transmission spectra at port 1 from 13.38 to 13.51 GHz.

Based on the above analyses, we further construct a two-port structure consisting of a straight ACB sandwiched waveguide and a PCRR, as shown in Figure 4a. The waveguide channel contains only one layer of lattice C. The distance between the PCRR and the straight waveguide is 2*a*. The lower part PC (i.e., lattice A) is applied with −*H* to support the pseudospin-down state propagating leftwards in the straight waveguide. The input and output ports are marked as $P_{0}$ and $P_{1}$, respectively. A pseudospin-down source (S−) marked by a black star is placed on the right side of $P_{0}$. Due to the coupling effect between the waveguide and the PCRR, the resonant frequencies are slightly changed. For example, the typical resonant frequencies at 13.394 GHz in a pure PCRR shift to 13.395 GHz now, and their $E_{z}$ field distributions are shown in Figure 4b,c. Obviously, for the case of 13.395 GHz, the EM wave can be easily coupled into the PCRR and then return the waveguide to propagate leftwards unidirectionally. However, for the standing mode at 13.503 GHz, since it has weaker coupling efficiency than the travelling mode at 13.395 GHz, after the EM wave enters the PCRR, it is well confined in the PCRR and hardly goes back to the waveguide again. These results are consistent with our previous analysis. Figure 4d presents the transmission spectra within the bandgap. The transmittances for 13.395 and 13.503 GHz marked by two dots as travelling mode and standing mode, respectively, also verify the previous analyses.

## 3. Results

### 3.1. Controllable Four-Port ADF

**Figure 5:**
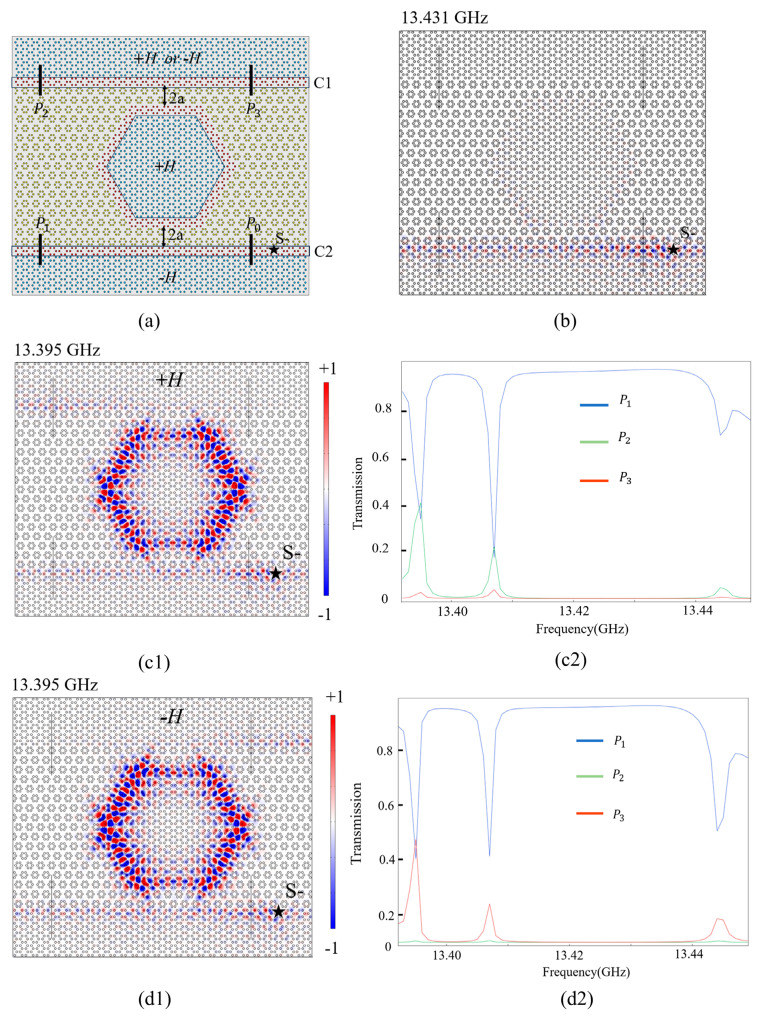
(**a**) Schematic diagram of the four-port ADF. (**b**) $E_{z}$ field distributions of the non-resonant frequency at 13.431 GHz. (**c1**,**c2**) $E_{z}$ field distributions of the travelling mode at 13.395 GHz and transmission spectra within the bandgap of 13.392–13.449 GHz when +*H* is applied to lattice A above C1. (**d1**,**d2**) $E_{z}$ field distributions and transmission spectra while +*H* is changed to −*H*.

Now, we proceed to study the working mechanism of a controllable topological ADF. Figure 5a shows the schematic diagram of the ADF which is composed of one PCRR and two straight waveguides. For convenience, we define the lower and upper straight waveguides as the bus and dropping channels (denoted by C2 and C1), respectively. The input port is $P_{0}$, and the output ports are $P_{1}$, $P_{2}$, and $P_{3}$. The regions of lattice A below C2 and inside the PCRR are applied with −*H* and +*H*, respectively. While for lattice A above C1, we can apply +*H* or −*H* to control the EM wave to exit in different dropping ports ($P_{2}$ or $P_{3}$). A pseudospin-down source (S−) denoted by a black star is placed in the bus channel near $P_{0}$.

The working mechanism of the controllable ADF is explained as follows. Figure 5b shows the simulation result for a non-resonant frequency at 13.431 GHz. Due to the non-resonant property, the EM wave propagates along C1 and does not couple with the PCRR, leading to total transmission from $P_{0}$ to $P_{1}$. When +*H* is applied to lattice A above C1, the $E_{z}$ field distribution at 13.395 GHz and the transmission spectra of three output ports within the bandgap are calculated, and they are shown in Figure 5(c1,c2). The incoming EM wave from $P_{0}$ is divided into two parts. One still transmits in C2 and exports from $P_{1}$, while the other is coupled with the PCRR and coupled into C1, so it ends up exporting from $P_{2}$. Similarly, Figure 5(d1,d2) show the results when the EMF applied to lattice A above C1 is reversed from +*H* to −*H*. The EM wave is also divided into two parts. One part still exits from $P_{1}$, while the other part is coupled to exit from $P_{3}$ instead of $P_{2}$, meaning that the dropping energy can be switched to exit from different ports by controlling the direction of the EMF applied to lattice A above C1. In other words, the topological waveguide-resonator system can be considered to be a topological ADF because it can easily add or remove signals to the output ports of waveguides.

**Figure 6:**
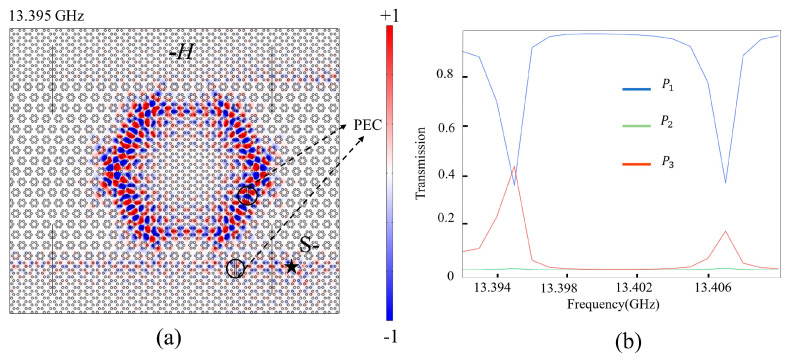
(**a**,**b**) $E_{z}$ field distribution at 13.395 GHz and transmission spectra when PEC defects are introduced into the waveguide and PCRR (−*H* is applied to lattice A above C1).

In addition, we investigate the robustness of the ADF by introducing two perfect electronic conductor (PEC) defects into the structure. One PEC is placed in the C2 channel, while the other is placed inside the PCRR, as denoted in Figure 6a. We apply −*H* for lattice A above C1. The $E_{z}$ field distribution at 13.395 GHz and transmission spectra of 13.392–13.412 GHz are shown in Figure 6a,b. Obviously, the EM wave bypasses the PECs to propagate forwards, only causing local phase change, but it has almost no influence on the transmittances at $P_{1}$, $P_{2}$, and $P_{3}$. From Figure 6b, one can find that the transmission spectra are almost the same as that without defects in Figure 5(d2). These results prove that the structure has strong robustness against PEC defects, which provides excellent tolerance for the fabrication of ADF.

### 3.2. Power Ratio Analyses of the ADF

**Figure 7:**
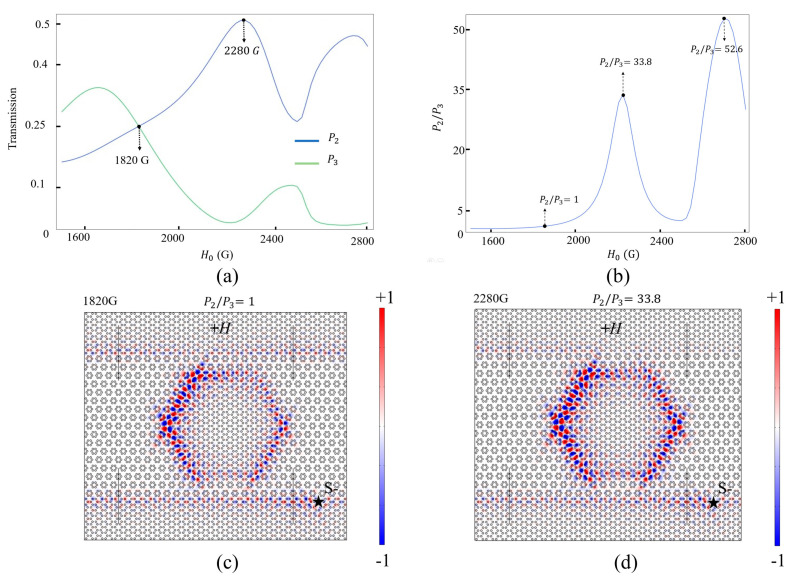
(**a**) Transmission spectra of the power output at $P_{2}$ and $P_{3}$ when +*H* = 1500~2800 G; (**b**) the power ratio of $P_{2}/P_{3}$ when +*H* = 1500~2800 G; (**c**,**d**) $E_{z}$ field distributions for $P_{2}/P_{3}$ = 1:1 and 33.8 at +*H* = 1820 and 2280 G, respectively.

In this section, we investigate the relationship between the power outputs and the strength of the EMF when the ADF structure serves as a power splitter. When +*H* > 2900 G, the transmittances at $P_{1}$, $P_{2}$, and $P_{3}$ will sharply decrease so that the strength of the EMF +*H* is limited from 1500 to 2800 G. Figure 7a shows the relationship curves between the transmittance ($P_{2}$ and $P_{3}$) and +*H* for the travelling mode at 13.395 GHz. Figure 7b further shows the power ratio of $P_{2}/P_{3}$ as +*H* increases from 1500 to 2800 G. The three black arrows denote the special points at different magnetic fields. As shown in Figure 7a, the blue curve for the power output from $P_{2}$ has two transmittance peaks of 51.1% and 47.3%, which correspond to the two valleys of the red curve for that from $P_{3}$. The power ratios of these two special points are 33.6 and 52.6, which are achieved for +*H* = 2280 and 2740 G, respectively. Additionally, there exists another special power ratio of $P_{2}/P_{3}$ = 1:1 when *H* = +1820 G, which means that the power of the EM wave is evenly split into $P_{2}$ and $P_{3}$ of the dropping channel. The maximum power ratio of $P_{2\;}/{\;P}_{3}\;$= 52.6 appears at +*H* = 2740 G. For example, Figure 7c,d present the *E_z_* field distributions for $P_{2\;}/{\;P}_{3}$ = 1:1 and 33.8 at +*H* = 1820 and 2280 G, respectively. Therefore, we have found a power splitter with a large available range of power ratio from near 0 to 52.6 by controlling the strength of the EMF.

## 4. Conclusions

In conclusion, we have designed a controllable hexagonal ADF constructed by topological MOPC waveguides and a PCRR. Due to the combined actions of broken time-reversal symmetry (TRS) and an analogous quantum spin Hall (QSH) effect, the MOPC waveguide supports the pseudospin-field-dependent state, while the PCRR possesses travelling and standing modes. Based on these properties, a topological four-port ADF is designed. The output port of the ADF can be altered by changing the direction of the EMF. Furthermore, the power ratio of the ADF ranging from near 0 to 52.6 is achieved by manipulating the strength of the EMF. The designed structure has strong robustness against obstacles. These results provide feasible ideas for constructing high-performance topological optical devices in wavelength division multiplexing systems.

## Data Availability

The data presented in this study are available on request from the corresponding author.

## Supplementary Materials

The following supporting information can be downloaded at: https://www.mdpi.com/article/10.3390/nano14110919/s1, Figure S1: The $\;E_{z}$ field distributions of the travelling mode (13.394 GHz) and standing mode (13.504 GHz) when its phase varies from 0 to 2π.

## Appendix A

**Table A1:** The setting details for simulations by COMSOL MULTIPHYSICS.

Precision	Coordinate system selection	Global coordinate system
	Boundary selection	Scattering boundary selection
	Orthonormal block limit	10,000,000
	Pivoting perturbation	1.0 × 10^−8^
Number of iterations	Maximum number of eigenvalue iterations	300
	Number of iterations in control entities	8
Details of spatial mesh	Spatial mesh	Free triangular mesh
	Predefined	Finer
	Maximum element size	0.00766 m
	Minimum element size	2.59 × 10^−5^ m
	Curvature factor	0.25
