# Supplementary figures and images for: Controllable Pseudospin Topological Add-Drop Filter Based on Magnetic–Optical Photonic Crystals
Source: Nanomaterials (Basel). 2024 May 23;14(11):919. doi: 10.3390/nano14110919 (PMC11173621; doi:10.3390/nano14110919)

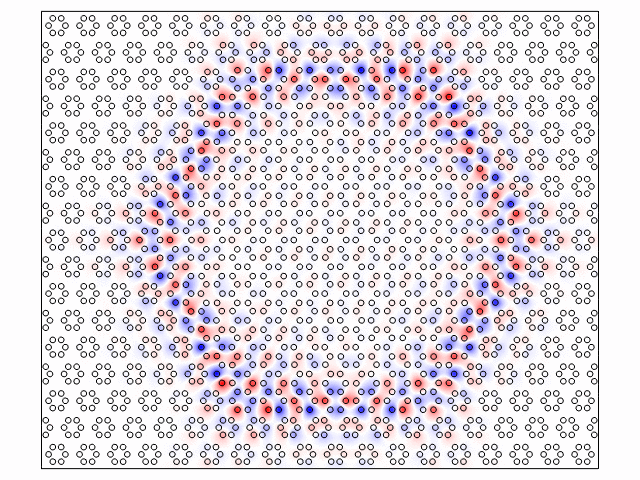

Supplement: Supplementary file 1 [file nanomaterials-14-00919-s001.zip › 13.503 GHz standing mode.gif]

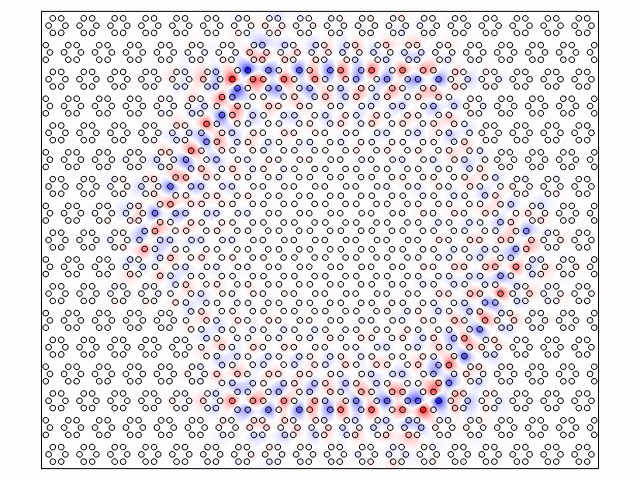

Supplement: Supplementary file 1 [file nanomaterials-14-00919-s001.zip › 13.394 GHz traveling mode.gif]
